# Cross-modal cell nucleus point clouds non-rigid registration for multiscale brain structure analysis

**DOI:** 10.1093/bib/bbaf631.029

**Published:** 2025-12-12

**Authors:** Yanan Lv, Jiangduo Liu, Xi Chen, Hua Han

**Affiliations:** Institute of Automation, Chinese Academy of Sciences, Beijing 100190, China; School of Artificial Intelligence, School of Advanced Interdisciplinary Sciences, University of Chinese Academy of Sciences, Beijing 100190, China; Institute of Automation, Chinese Academy of Sciences, Beijing 100190, China; School of Artificial Intelligence, School of Advanced Interdisciplinary Sciences, University of Chinese Academy of Sciences, Beijing 100190, China; Institute of Automation, Chinese Academy of Sciences, Beijing 100190, China; School of Artificial Intelligence, School of Advanced Interdisciplinary Sciences, University of Chinese Academy of Sciences, Beijing 100190, China; Institute of Automation, Chinese Academy of Sciences, Beijing 100190, China; School of Artificial Intelligence, School of Advanced Interdisciplinary Sciences, University of Chinese Academy of Sciences, Beijing 100190, China

## Abstract

**Background:**

Multiscale information integration is essential for a comprehensive understanding of brain structure and function ^[1]^. Optical microscopy provides mesoscopic information on brain region distribution, neuronal projections, and functional activity patterns, while electron microscopy offers microscopic details, such as cell morphology and synaptic connectivity. Aligning cell nucleus information from these two modalities is therefore critical for linking global tissue organization with cellular-level mechanisms. However, fundamental differences in imaging principles and data characteristics make cross-modal cell nucleus point clouds registration highly challenging ^[2]^. Variations in point density, inconsistent noise levels, and complex nonlinear deformations often prevent traditional methods from achieving the accuracy and biological plausibility required in neuroscience research.

**Method:**

We propose a non-rigid registration strategy for cross-modal cell nucleus point clouds. The method adopts a multi-level block correspondence scheme to achieve consistent alignment across local and global scales, while integrating neighborhood constraints and a bidirectional weighting mechanism to mitigate the influence of outliers and data sparsity.

**Experiment:**

Experimental results demonstrate that this strategy significantly improves registration accuracy and robustness while preserving structural continuity. This work provides technical support for cross-modal neural tissue mapping and demonstrates the potential of multiscale information integration to advance brain connectomics and neurological disease research.

**References:**

[1] Shapson-Coe A, Januszewski M, Berger D R, et al. ‘A petavoxel fragment of human cerebral cortex reconstructed at nanoscale resolution’ [J]. Science, 2024, 384(6696).

[2] Huang X, Mei G, Zhang J. ‘Cross-source point cloud registration: Challenges, progress and prospects’ [J]. Neurocomputing, 2023, 548: 126383.

